# Cardiac Biomarkers Predicting MACE in Patients Undergoing Noncardiac Surgery: A Meta-Analysis

**DOI:** 10.3389/fphys.2018.01923

**Published:** 2019-01-18

**Authors:** Li-Jun Zhang, Na Li, Yang Li, Xian-Tao Zeng, Mei-Yan Liu

**Affiliations:** ^1^Department of Cardiology, Beijing Anzhen Hospital, Capital Medical University, Beijing, China; ^2^Center for Evidence-Based and Translational Medicine, Zhongnan Hospital of Wuhan University, Wuhan, China; ^3^Center for Evidence-Based and Translational Medicine, Wuhan University, Wuhan, China

**Keywords:** cardiac biomarkers, cardiac risk factors and prevention, acute coronary syndromes, noncardiac surgery, meta-analysis

## Abstract

**Objective:** The present meta-analysis was aimed to systematically evaluate the effectiveness and accuracy of brain natriuretic peptide (BNP), cardiac troponin (cTn), high sensitive C reactive protein (hs-CRP) and CRP for predicting postoperative major adverse cardiovascular events (MACE) in patients undergoing noncardiac surgery.

**Methods:** A total of 26 relevant studies with 7,877 participants were collected from five databases, namely PubMed, Embase, China National Knowledge Infrastructure (CNKI), CQVIP and the Wanfang Database until August 10, 2018. And the Review Manager Version 5.3 and Stata/SE 12 software were used for data syntheses in the meta-analysis.

**Results:** Strong relationships of BNP/NT-proBNP, cTnI/cTnT and hs-CRP with MACE were detected in patients undergoing noncardiac surgery, and the five biomarkers all increased the risk of MACE. Compared to normal levels, elevated BNP/NT-proBNP could increase the MACE risk by almost 4-fold [RR:3.92, 95%CI: 3.23–4.75, *P* < 0.001]; elevated BNP corresponded to a 4.5-fold risk [RR:4.57, 95%CI: 3.37–6.20, *P* < 0.001]; elevated NT-proBNP led to a 3-fold higher risk [RR:3.48, 95%CI: 2.71–4.46, *P* < 0.001]. Comparing with normal levels of cTnI/cTnT, increased cTnI/cTnT was associated with nearly 5-fold more higher risk of MACE [RR:5.52, 95%CI: 4.62–6.58, *P* < 0.001]; elevated cTnI faced a 5-fold risk [RR:5.21, 95%CI: 3.96–6.86, *P* < 0.001]; elevated cTnT resulted in nearly 6-fold higher risk [RR:5.73, 95%CI: 4.55–7.22, *P* < 0.001]. The elevation of hs-CRP was associated with nearly 4-fold higher risk of MACE in comparison with normal concentration [RR:3.73, 95%CI: 2.63–5.30, *P* < 0.001].

**Conclusion:** According to the results of our meta-analysis, the elevations of BNP/NT-proBNP, cTnI/cTnT, and hs-CRP, pre-operation or post-operation immediately, can predict much higher risk of postoperative MACE in patients undergoing noncardiac surgery.

## Introduction

Although the rate of perioperative events shows a declining tendency in the past 30 years, a consequence of the developments in anesthesiology and surgical techniques, cardiovascular complications still represent a leading cause of perioperative mortality in individuals receiving large noncardiac surgery (Devereaux et al., 2005). At the moment, the main method to assess adverse cardiovascular events lies in the evaluation of various risk factors, and predicted high-risk patients need undergo more detailed examinations during perioperative period, such as echocardiography and cardiac angiography. Therefore, it is important to formulate a cost-effective, convenient and accurate method for preoperative evaluation, risk stratification and optimization prior surgery, and the use of cardiac biomarkers has been suggested for this role. Biomarkers may help to reduce the morbidity rate of major adverse cardiovascular event (MACE, including death, stroke, myocardial infarction, and need for coronary artery bypass graft or percutaneous coronary intervention) during perioperative and postoperative period.

Cardiac biomarkers, such as brain natriuretic peptide (BNP) and amino-terminal pro-hormone of brain natriuretic peptide (NT-proBNP), cardiac troponins (cTn), high sensitive C reactive protein (hs-CRP) and CRP, are currently considered to play a pivotal role in evaluating treatment strategy and prognosis of different cardiac diseases (McMurray et al., 2012; Clerico et al., 2014; Thygesen et al., 2018). European Society of Cardiology and European Society of Anesthesiology guidelines for preoperative cardiac risk assessment have recommended that preoperative BNP or NT-proBNP measurement should be considered in high-risk patients undergoing noncardiac surgery (Thygesen et al., 2018). In addition, BNP would increase during exercise in patients without heart failure and healthy individuals, resulting from transient myocardial wall stress, cardiomyocyte metabolic changes, and neuroendocrinology response (Hamasaki, 2016).

In recent years, a large number of studies have verified the value of preoperative BNP or NT-proBNP, cTnI/cTnT, and hs-CRP/CRP for cardiovascular risk prediction in patients undergoing noncardiac surgery (McMurray et al., 2012; Clerico et al., 2014). The aim of our study was to evaluate the correlation between preoperative cardiac biomarkers and perioperative MACE in noncardiac surgery patients through the method of meta-analysis, with the hope of providing better evidence for clinical application.

## Methods

### Search Strategy

We methodically searched the databases of PubMed, Embase, China National Knowledge Infrastructure (CNKI), VIP China Science, and Wanfang with the combination of the following search keywords: “b-type natriuretic peptide” or “Natriuretic Peptide, Brain” or “high-sensitivity C reactive protein” or “hs-CRP” or “C reactive protein” or “CRP” or “Troponin.” “cardiac events” or “cardiovascular events” or “major adverse cardiac events,” and “surgery.” Publication languages were limited to English and Chinese only. Literature was restricted to those published before December 09, 2018. References of relevant articles were further screened to identify additional eligible studies. The principal authors of reports lacking essential data would be contacted to obtain detailed information for our meta-analysis.

### Data Extraction

Relevant articles were selected independently by two experienced investigators based on the following criteria: (1) participants' age ≥ 18 years, (2) with a cohort study design, (3) stating the level of cardiac biomarkers detected before or immediately after operation, (4) study subjects only receiving noncardiac surgery, (5) providing sufficient information for the calculation of risk ratio (RR), and (6) offering complete text.

Quality assessment: All of the included articles were reviewed in detail. Essential information was independently extracted by two experienced investigators, and discrepancies over these data between the two investigators were resolved through discussion to reach a final consensus. When two or more articles used the same group of original population data, only the article with the largest sample size was included in this meta-analysis. The Newcastle-Ottawa Scale (NOS) 24 was used to assess the quality of all included articles (Stang, 2010). This Scale has three parts: selection, comparability, and exposure, containing eight items. The total score ranges from zero to nine stars. We classified articles with seven to nine stars as high quality, those with five to six stars as medium quality, and those with zero to four stars as poor quality. And articles with poor quality were excluded.

### Detail Information About MACE

Patients undergoing noncardiac surgeries are at high risk of MACE, which is a significant clinical and economical challenge. MACE mentioned in this meta-analysis included heart failure, acute coronary syndrome, atrial fibrillation, paroxysmal supraventricular tachycardia, ventricular tachycardia, angina pectoris, acute myocardial infarction, thromboembolic events, deep vein thrombosis, acute renal failure, transient ischemic attack, cardiac death, all-cause mortality, major arrhythmia, unstable angina, stroke, cardiac revascularization procedure.

### Statistical Analysis

Data were analyzed using Review Manager (RevMan) Version 5.3 (The Nordic Cochrane Center, Copenhagen, Denmark) and Stata/SE 12 (StataCorp., College Station, Texas). Risk ratio (RR) with 95% confidence (95% CI) was calculated to measure effect size. Heterogeneity was evaluated with Cochran's Q-test (DerSimonian and Laird, 1986) and I^2^ statistic (Melsen et al., 2014), and would be considered as insignificant to moderate when I^2^ = 0–50% while as statistically significant when I^2^ > 50%. Random-effects model would be used for calculating pooled effect size when heterogeneity was significant (*P* < 0.10 or I^2^ > 50%), while fixed-effects model would be utilized in the absence of significant heterogeneity (*P* > 0.10 or I^2^ < 50%). Considering the discrepancies in measured cardiac biomarkers across the studies, stratified analyses were performed in terms of biomarkers' types and methods for biomarker measurement. In addition, sensitivity analyses were performed via excluding studies one at a time (Tobias, 1999). Funnel plots were adopted to detect publication bias (Begg and Mazumdar, 1994). P-values were two-sided, and < 0.05 was considered statistically significant.

## Results

### Literature Selection

**Figure 1:**
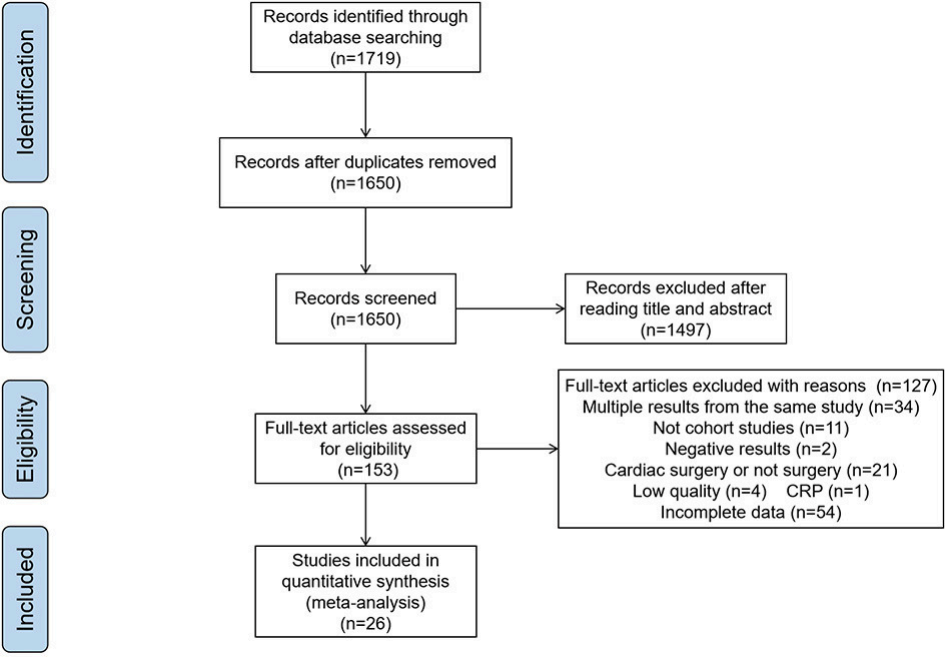
The flow diagram of the meta-analysis.

Initially, 1,719 publications were obtained from the databases. After removing duplicates and reviews, 1,650 articles were left. In the examinations on titles and abstracts, 1,497 articles were excluded. Of the remaining 153 papers, 26 articles were considered to be eligible for our study after reading the full texts, and the other 127 articles were excluded for the following reasons: multiple results from the same study (*n* = 34), not cohort studies (*n* = 11), negative results (*n* = 2), cardiac surgery (*n* = 21), low quality (*n* = 4), incomplete data (*n* = 54), only 1 article for CRP, so we excluded it (*n* = 1). (Figure 1)

Study Characteristics: A total of 7,877 participants were recruited in the eligible articles Among them three articles (Oscarsson et al., 2009b; Stone et al., 2014; Kopec et al., 2017) reported two biomarkers, eleven studies detected an association between BNP/ NT-proBNP and MACE (Feringa et al., 2007; Rajagopalan et al., 2008, 2011; Oscarsson et al., 2009a,b; Vetrugno et al., 2012; Cagini et al., 2014; Stone et al., 2014; Kim et al., 2016; Long et al., 2016; Kopec et al., 2017), fourteen reported an association between cTn and MACE (Lopez-Jimenez et al., 1997; Oscarsson et al., 2004, 2009b; Gibson et al., 2006; Ausset et al., 2008; Dawson-Bowling et al., 2008; Chong et al., 2009; Winkel et al., 2010; Biccard et al., 2012; Shroff et al., 2012; Gillmann et al., 2014; Kopec et al., 2017; Golubović et al., 2018; Kim et al., 2018), and four revealed a relationship between hs-CRP and MACE (Owens et al., 2007; Martins et al., 2011; Scrutinio et al., 2011; Stone et al., 2014; Table 1). The quality evaluation according to NOS of each selected article was also shown in Table 1. The studies included in this meta-analysis were all cohort studies, including 1 nested cohort study (Kopec et al., 2017), 5 retrospective studies (Shroff et al., 2012; Stone et al., 2014; Kim et al., 2016, 2018; Long et al., 2016) and others were prospective studies. Meanwhile, Kim reported NT-proBNP, Kopec reported NT-proBNP and cTnT, Long and Stone reported BNP, Shroff and Kim reported cTnI, and Stone also reported hs-CRP.

**Table 1:** Characteristics of articles in the meta-analysis.

**References**	**Case**	**Operation type**	**Testing index**	**Acquisition time**	**Threshold**	**Measurement**	**Follow-up time**	**MAC**	**NOS**
Ausset et al., 2008	87	Hip surgery	cTnI	The first three postoperative days	>0.08ng/ml	Ortho Vitros ECi; Ortho-Clinical Diagnostics	during hospital and 1 year	cardiac death, MI,HF, ACS	7
Biccard et al., 2012	788	Vascular surgery	cTnI	Immediately,1,2,3,7 days postoperation	–	Advia Centaur Xp (Siemens Healthcare)	30 d	Death or cTnI equal to or higher then reference level	7
Cagini et al., 2014	294	Pneumonectomies, lobectomies, anatomical segmentectomies or wedge resections	BNP	Before and 1,4 days postoperation	>118.5pg/ml	–	24h	AF,PSVT,VT, AMI,UP, ARF, TIA,DVT, thromboembolic events	6
Chong et al., 2009	102	Emergency orthopedic surgery	cTnI	Pre-operation and on days 1, 2, and 3 post-operation	>0.3 mcg/l	Architect STAT Troponin I assay (Abbott Diagnostics)	In hospital / 1 year	MI, HF, AF, major arrhythmia	7
Dawson-Bowling et al., 2008	108	Fractured of neck and femur	cTnT	Admission day 1–2 postoperation	>0.03ng/ml	–	In hospital	MI, HF, unstable angina, major arrhythmias requiring treatment	5
Feringa et al., 2007	335	Abdominal aortic repair, lower extremity revascularization	NT-proBNP	Before surgery	≥319 ng/L	Electrochemiluminescence immunoassay	14 ± 6 year	Cardiac death, MI	8
Gibson et al., 2006	44	Below knee amputation	cTnT	Before surgery	>0.3ng/ml	Elecsys 2010 (Roche Diagnostics)	6 weeks postoperation	Non-fatal MI, cardiac death	9
Gillmann et al., 2014	455	Elective open aortic, peripheral vascular, or carotid surgery	cTnT	Before and 24 h postoperation	≥17.8ng/L	Immunoassay	30 days	Cardiovascular death, MI, ischemia	7
Golubović et al., 2018	79	Abdominal, thoracic, orthopedic, and vascular surgery	cTnT	48h prior to surgery	>7.5ng/L	Immunoluminometric electrochemiluminescence immunoassay	14 days	HF, MI, all-cause mortality	6
Kim et al., 2016	506	Non-cardiac surgery of elderly patients with normal left ventricular systolic function	NT-proBNP	Before surgery	≥425.3pg/ml	Elecsys 2010 (Roche Diagnostics)	30 d	Cardiac death, AMI, HF, stroke, AF	7
Kim et al., 2018	262	Hip fracture surgery	cTnI	Before surgery	>6.5ng/L	Immunoassay analyzer	90 days	All-cause deaths, HF, AF, MI	7
Kopec et al., 2017	572	Major noncardiac surgery,	cTnT NT-proBNP	Before surgery	≥14 ng/L/ >108ng/L	Elecsys 2010 (Roche Diagnostics)	The first 3 days after surgery	MI	7
Long et al., 2016	1120	Primary total knee arthroplasty	BNP	Before surgery	≥822.5pg/ml	Elecsys electrochemiluminescence immunoassay (ECLIA)	≥2 years	AF, AHF, MI	6
Lopez-Jimenez et al., 1997	772	Noncardiac surgery without clinical evidence of severe ischemia	cTnT	In the recovery room and during the next 2 days	>0.1ng/ml	–	6 months	Cardiac deaths, nonfatal MI, unstable angina	8
Martins et al., 2011	101	Aortic and peripheral artery revascularizations	Hs-CRP	before surgery	>100 mg/L	VITROS Chemistry Products (Ortho-Clinical Diagnostics)	Until discharge	AMI, noncardiac death, AF, HF, ARF	8
Oscarsson et al., 2009b	186	Emergent or urgent non-cardiac surgery	cTnT NT-proBNP	Within 1 h before surgery	0.06 μg/l/>1800 pg/ml	Stratus CS Acute Care^TM^ Diagnostic System (Dade International Holding GmBH)	30 d	AMI, cardiovascular death	8
Oscarsson et al., 2009a	69	Unscheduled hip surgery	NT-proBNP	1 h before surgery	≥3984ng/ml	Stratus CS Acute Care^TM^ Diagnostic System (Dade International Holding GmBH)	30 days after operation	AMI, cardiac events	8
Oscarsson et al., 2004	161	Gynecology, orthopedics, urology, vascular surgery	cTnT	5−7 h postoperation	≥0.02 ng/ml	ECLIA on Elecays 2010 analyzer	1 year	AMI, coronary intervention	8
Owens et al., 2007	91	Autogenous lower extremity revascularization	Hs-CRP	Immediately before surgery	>5 mg/L	BD Diagnostics	342 days (range, 36 to 694)	Stroke, MI, cardiac revascularization procedure, death fromAny cause	8
Rajagopalan et al., 2008	136	Subcritical limb ischemia or abdominal aortic aneurysm (AAA) repair	NT-proBNP	Before surgery	>308pg/ml	Elecsys 2010 (Roche Diagnostics)	2 days	Revascularization procedure, or death from any cause	6
Rajagopalan et al., 2011	136	Open abdominal aortic aneurysm (AAA) repair and aorto-femoral and infrainguinal revascularization for critical limb ischaemia	NT-proBNP	The day before surgery and on the first postoperative day after surgery	≥359pg/ml	Electrochemiluminescence sandwich immunoassay (ECLIA)	2 years	Death due to an obvious cardiac cause or coronary revascularization	7
Scrutinio et al., 2011	239	Elective carotid or peripheral lower extremity revascularization or aortic surgery	Hs-CRP	On the preoperative day	>3.2 mg/L	Siemens Healthcare Diagnostics	30days	cTn-I elevation above the decision limit of 0.15 g/L, death, ACS, stroke, AHF	8
Shroff et al., 2012	376	Kidney transplantation	cTnI	Before surgery	≥0.04 ng/ml	Vitros, Ortho Clinical Diagnostics	In hospital	Death, MI, arrhythmia or HF	8
Stone et al., 2014	118	Lower extremity, EVT	hs-CRP/ BNP	Before surgery	>0.8mg/dl/ >100pg/ml	Immunometric assay	1year, 2 years	Stroke, MI, death	6
Vetrugno et al., 2012	227	Elective prosthesis orthopedic surgery	BNP	Before surgery	>39 pg/dl	Bayer ADVIA Centaur™	In hospital	AF, AHF, non-fatal/fatal MI	7
Winkel et al., 2010	513	Vascular surgery	cTnT	Before surgery	>0.1ng/ml	Electrochemiluminescence immunoassay (TropT version 2, Roche Diagnostics GmbH).	Postoperative days 1,3, 7, 30 2 years	Cardiac death, MI	8

*NOS, Newcastle-Ottawa Scale; cTnI, cardiac troponin I; BNP, brain natriuretic peptide; NT-proBNP, amino-terminal pro-hormone of brain natriuretic peptide; HF, heart failure; AHF, acute heart failure; ACS, acute coronary syndrome; AF, atrial fibrillation; PSVT, paroxysmal supraventricular tachycardia; VT, ventricular tachycardia; UP, angina pectoris; MI, myocardial infarction; AMI, acute myocardial infarction; DVT, deep vein thrombosis; ARF, acute renal failure; TIA, transient ischemic attack*.

### BNP/NT-proBNP and MACE

**Figure 2:**
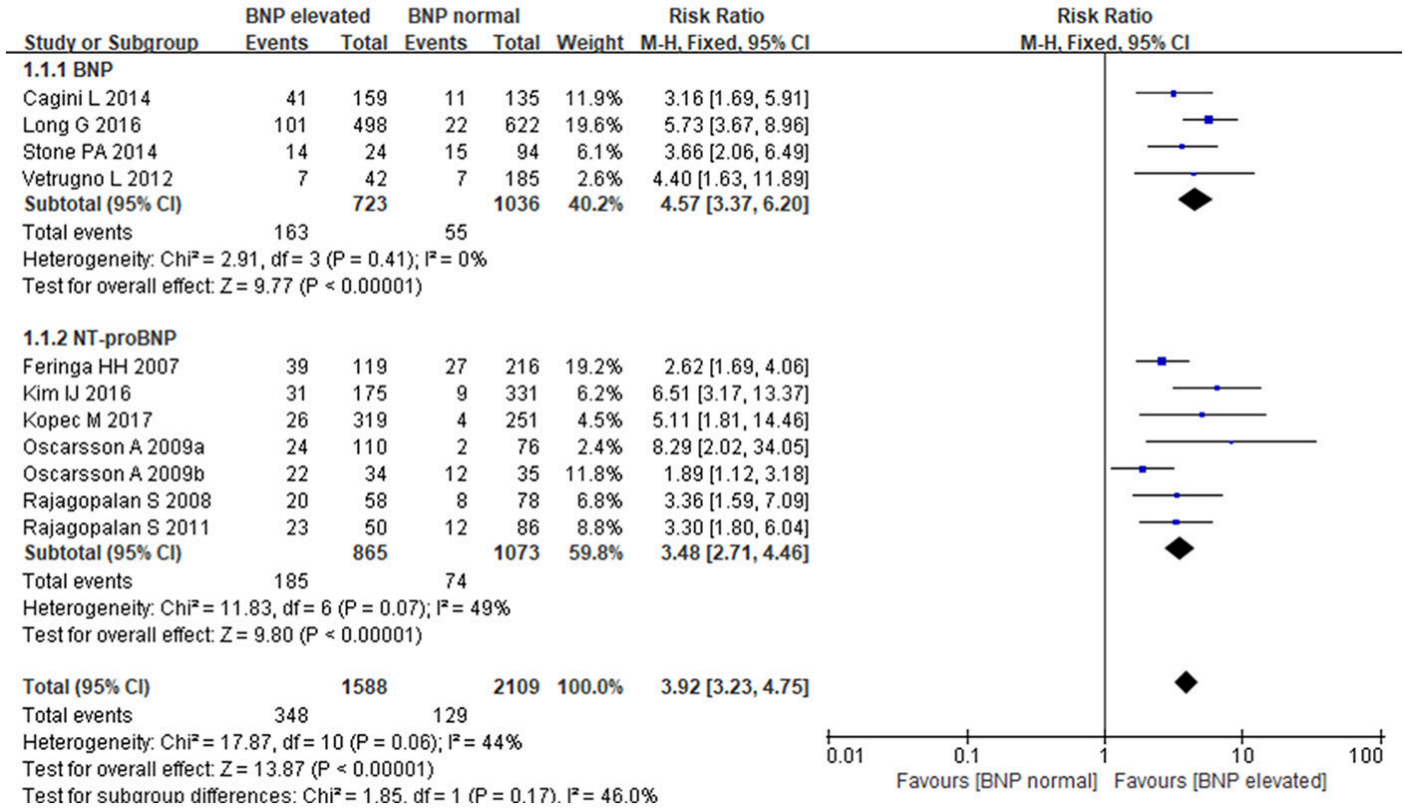
The association between BNP/NT-proBNP and MACE.

The fixed-effects model was utilized to pool summary effects for BNP/NT-proBNP (*P* = 0.06, I^2^ = 44%). Strong relationship was detected between BNP/NT-proBNP and MACE in patients undergoing noncardiac surgery, and BNP and NT-proBNP both increased MACE risk. Comparing with normal BNP/NT-proBNP, elevated BNP/NT-proBNP was associated with an almost 4-fold higher risk of MACE [RR: 3.92, 95%CI: 3.23–4.75, *P* < 0.001]. Subgroup analysis showed that elevated BNP increased MACE risk by 4.5-fold when compared to normal BNP [RR:4.57, 95%CI: 3.37–6.20, *P* < 0.001], and elevated NT-proBNP led to a more than 3-fold higher risk of MACE when compared to normal NT-proBNP [RR:3.48, 95%CI: 2.71–4.46, *P* < 0.001] (Figure 2). In the subgroup analysis of BNP regarding different study types, retrospective studies showed elevated BNP increased MACE risk by 5-fold when compared to normal BNP [RR:5.24, 95%CI:3.61–7.61, *P* < 0.001], prospective studies showed elevated BNP increased MACE risk by 3-fold when compared to normal BNP [RR:3.39, 95%CI:1.98–5.79, *P* < 0.001].

### cTnI/cTnT and MACE

**Figure 3:**
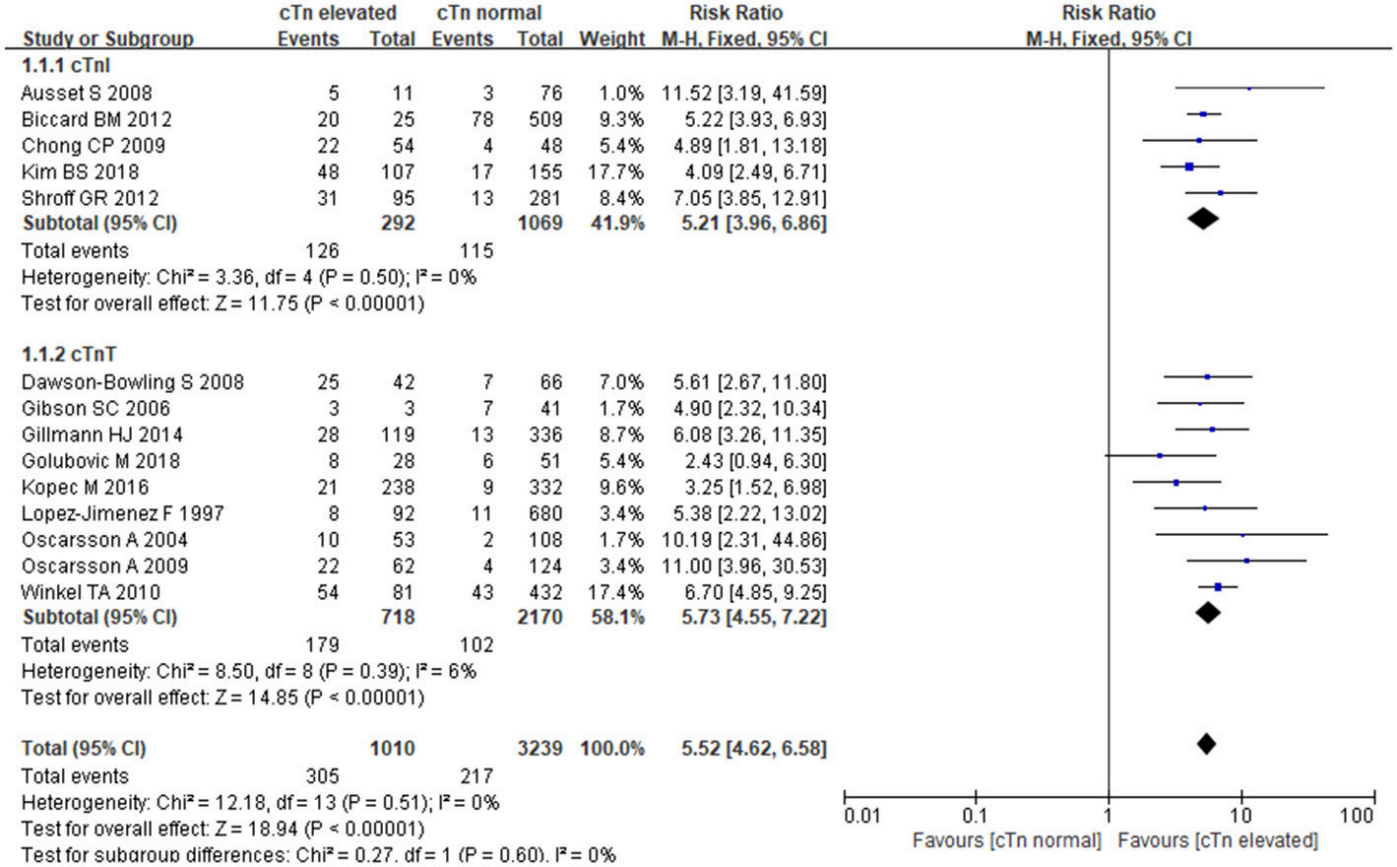
The association between cTnI/cTnT and MACE.

The fixed-effects model was employed for data pooling (*P* = 0.51, I^2^ = 0%). According to summarized results, cTnI, and cTnT could strongly increase the risk of MACE in patients undergoing noncardiac surgery. Comparing with normal cTnI/cTnT, the elevation of cTnI/cTnT was associated with nearly 5-fold more higher risk of MACE [RR:5.52, 95%CI: 4.62–6.58, *P* < 0.001]. In stratified analysis, elevated cTnI conferred a 5-fold risk [RR:5.21, 95%CI: 3.96–6.86, *P* < 0.001]; when compared to normal cTnI, and elevated cTnT resulted in nearly 6-fold higher risk when compared to normal cTnT [RR:5.73, 95%CI: 4.55–7.22, *P* < 0.001] (Figure 3). In the subgroup analysis of cTnI regarding different study types, retrospective studies showed elevated cTnI increased MACE risk by 5-fold when compared to normal cTnI [RR:5.49, 95%CI:3.76–8.03, *P* < 0.001], prospective studies showed elevated cTnI increased MACE risk by 9-fold when compared to normal cTnI [RR:5.04, 95%CI:3.45–7.37, *P* < 0.001].

### hs-CRP and MACE

**Figure 4:**
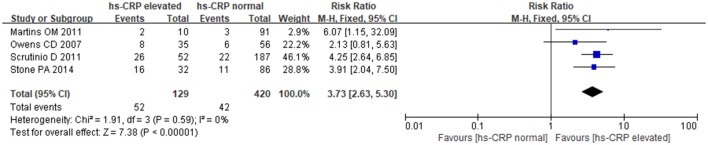
The association between hs-CRP and MACE.

The fixed-effects model was utilized for pooling data (*P* = 0.59, I^2^ = 0.0%). The results showed a strong relationship between hs-CRP and increased MACE in patients undergoing noncardiac surgery. Comparing with normal hs-CRP, the elevation of hs-CRP could increase MACE risk by nearly 4-fold [RR: 3.73, 95%CI: 2.63–5.30, *P* < 0.001] (Figure 4).

### Sensitivity Analysis

**Figure 5:**
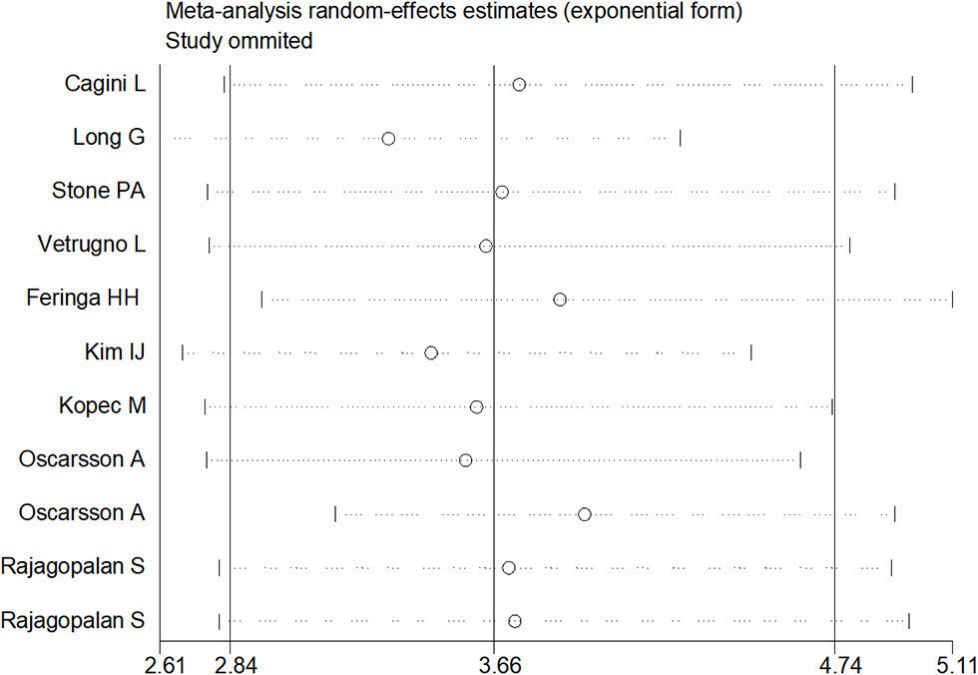
Sensitivity analysis in BNP and NT-proBNP.

**Figure 6:**
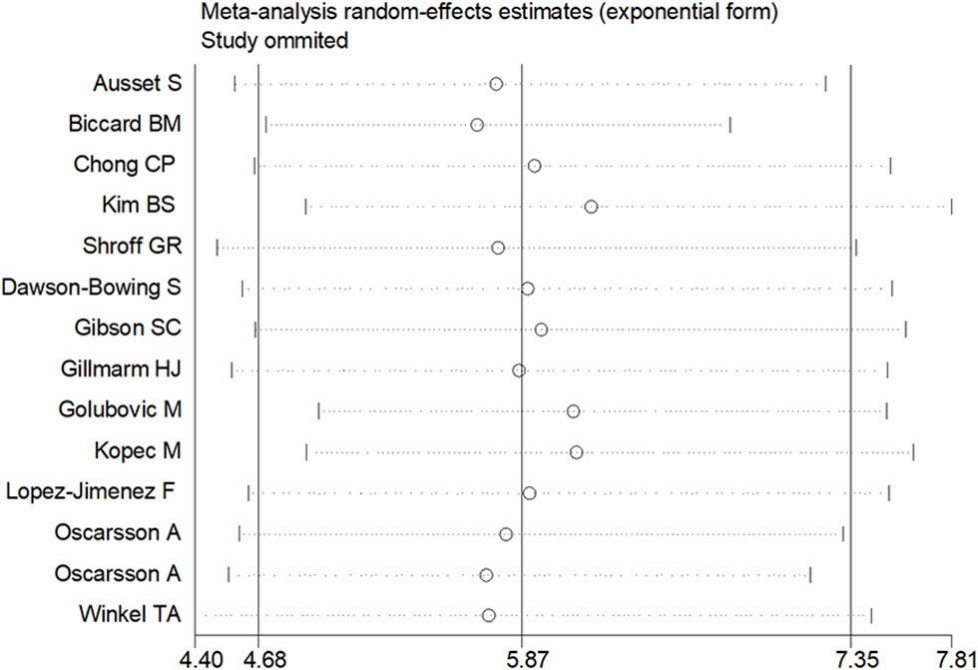
Sensitivity analysis in cTnI and cTnT.

Sensitivity analysis was performed through excluding a single study each time to detect the influence of individual datasets on pooled RRs. The analysis results for BNP and NT-proBNP suggested that the power of pooled RRs was decreased after excluding Long G's study (Long et al., 2016) while increased after excluding Feringa HH's (Feringa et al., 2007) or Oscarsson A's study (Oscarsson et al., 2009a). We considered the potential reasons for the analysis results included: (1) Long G's study was a retrospective and enrolled the most patients than other studies; (2) Oscarsson A's study had the fewest participants than other studies; (3) Compared with other studies, there was minimum difference of MACE incidence between BNP elevated group and BNP normal group in Feringa HH's study. Nonetheless, the RRs were not altered when removing any one of the other studies. The analysis for cTnI, cTnT and hs-CRP found no obvious alterations the final pooled RRs during the exclusion of any studies. The results from sensitivity analysis indicated that our results were robust (Figures 5, 6).

### Publication Bias

**Figure 7:**
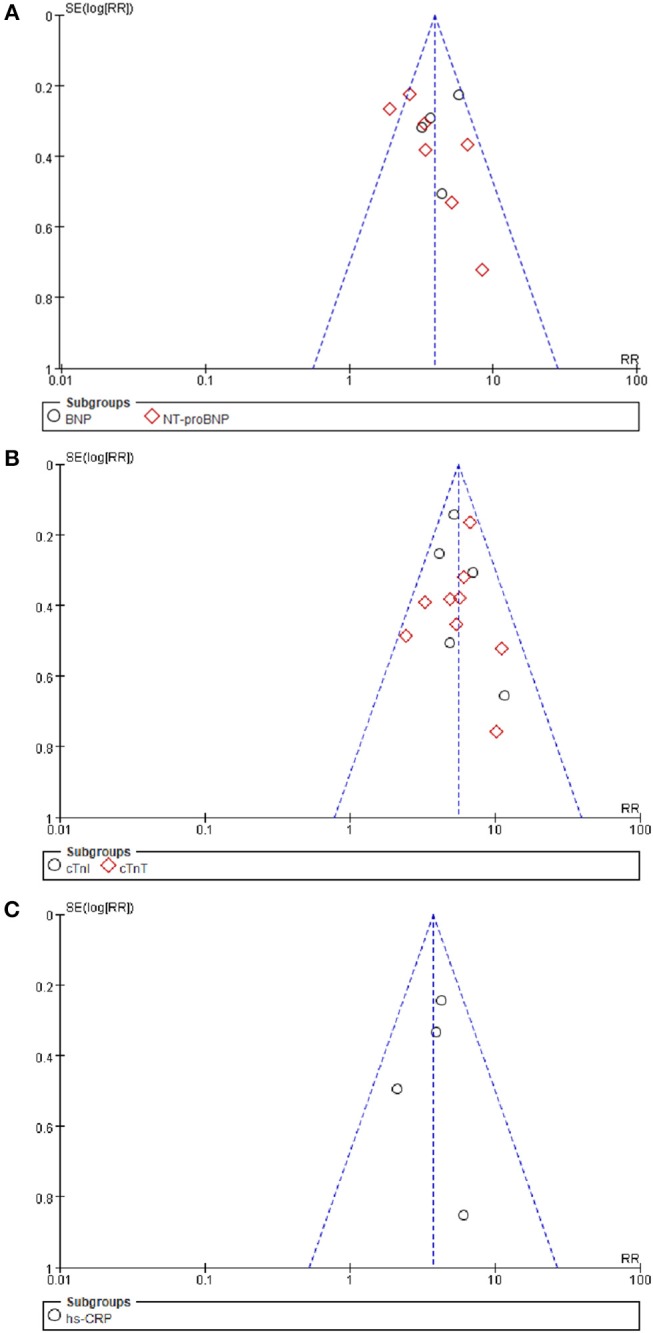
**(A–C)** Begg's funnel plot with pseudo 95% confidence limits.

The funnel plot for BNP/NT-proBNP, cTnI/cTnT, and hs-CRP showed no asymmetry, indicating publication bias was insignificant in this meta-analysis. Besides, such inference was further supported by statistical evidence from modified Begg's tests. Therefore, there was no significant publication bias between included studies in this meta-analysis (*P* = 0.39 for BNP/NT-proBNP; *P* = 0.23 for cTnI/cTnT; *P* = 0.96 for hs-CRP) (Figure 7).

## Discussion

In this meta-analysis, we found a strong relationship of BNP/NT-proBNP [RR:3.92, 95%CI: 3.23–4.75, *P* < 0.001] (Feringa et al., 2007; Rajagopalan et al., 2008, 2011; Oscarsson et al., 2009a,b; Vetrugno et al., 2012; Cagini et al., 2014; Stone et al., 2014; Kim et al., 2016; Long et al., 2016; Kopec et al., 2017), cTnI/cTnT [RR:5.85, 95%CI: 4.85–7.06, *P* < 0.001] (Lopez-Jimenez et al., 1997; Oscarsson et al., 2004, 2009b; Gibson et al., 2006; Ausset et al., 2008; Dawson-Bowling et al., 2008; Chong et al., 2009; Winkel et al., 2010; Biccard et al., 2012; Shroff et al., 2012; Gillmann et al., 2014; Kopec et al., 2017; Golubović et al., 2018; Kim et al., 2018), and hs-CRP [RR:3.73, 95%CI: 2.63–5.30, *P* < 0.001] (Owens et al., 2007; Martins et al., 2011; Scrutinio et al., 2011; Stone et al., 2014) with MACE in patients undergoing noncardiac surgery, and all of these biomarkers were positively related to the risk of MACE.

It has been reported that patients undergone noncardiac surgeries are at high risk of MACE due to multiple comorbidity (Kumar et al., 2001). Elderly patients after hip fracture surgery have higher mortality (Oscarsson et al., 2009a). Post-operative MI often occurs silently and come up with poor prognosis (Roberts and Goldacre, 2003). Almost one third of patients suffered myocardial ischaemia in the perioperative period of fractured neck of femur surgery (Roberts and Goldacre, 2003). Therefore, biomarkers detection could be a potential cardiac assessment for cardiac events.

Though different study types may cause certain bias, we found no significant difference caused by study types in this meta-analysis. All the studies included in this meta-analysis were cohort studies, including 1 nested cohort study (Kopec et al., 2017), 5 retrospective studies (Shroff et al., 2012; Stone et al., 2014; Kim et al., 2016, 2018; Long et al., 2016) and others were prospective studies. Meanwhile, Kim reported NT-proBNP, Kopec reported NT-proBNP and cTnT, Long and Stone reported BNP, Shroff and Kim reported cTnI, and Stone also reported hs-CRP. We made a subgroup analysis according to study type in BNP and cTnI. The results showed no difference in retrospective studies and prospective studies. In the sensitivity analyses of NT-proBNP, cTnT, and hs-CRP, we could see no significant difference was found after removing Kim, Kopec, and Stone's studies respectively.

Both NT-proBNP and BNP are derived from the cleavage of BNP precursor, but NT-proBNP has longer half-life, higher plasma concentration and more stable biological properties than BNP, though both are associated with a variety of heart diseases such as left ventricular dysfunction, valvular heart disease, and acute coronary syndrome (Weber and Hamm, 2006). BNPs are secreted from the myocardium in response to multiple stimuli such as ischaemia, myocardial stretch, inflammation and other neuroendocrine stimuli (Clerico et al., 2011). The level of BNP or NT-proBNP may provide guidance for preoperative preparation, especially for the assessment of ventricular systolic and diastolic function, and they even may reflect minor changes in ventricular function induced by transient myocardial ischemia. Recent studies have found lower BNP and NT-ProBNP levels in patients with heart failure with preserved ejection fraction (HFPEF) than those with reduced ejection fraction (HFREF) (Bishu et al., 2012). Moreover, Ishigaki T et al (Ishigaki et al., 2015) demonstrated the different implications of elevated BNP in HFPEF and HFREF, the former may be associated with high filling pressure, while the latter may be related with myocardial dysfunction. Meanwhile, plasma BNP levels can also reflect the risk and severity of coronary artery lesions in patients with stable coronary heart disease (Niizuma et al., 2009). Therefore, it is reasonable to choose BNP or NT- proBNP level as predictor for post-noncardiac surgery cardiovascular events. Lucio Cagini et al. (2014) demonstrated that increased BNP first days after thoracic surgery including pneumonectomies, lobectomies, anatomical segmentectomies or wedge resections, resulted in 3-fold higher risk of developing postoperative cardiopulmonary complications. In patients with hip fractures, Oscarsson et al. (2009a) found that the elevation of preoperative NT-proBNP was strongly connected with cardiac complications in perioperative period. Stone et al. (2017) had also proven the significant role of BNP in predicting MACE in patients undergoing carotid intervention, however, due to the incomplete data, we haven't included this paper. In consistent with the conclusions of previous studies, our meta-analysis unveils that both BNP and NT-proBNP are valuable predictors for MACE in postoperative period.

Troponin (Tn) is a structural protein of cardiac contractility and a part of the muscle calcium tropomyosin complex, showing a major role in skeletal and cardiac muscle contraction. There are three subunits in troponin complex: TnI, TnT, and TnC. Our study mainly focused on cTnI and cTnT for they are highly sensitive markers of myocardial injury. Three to six hour after the damage to cardiac muscle cells, troponins can be detected in patient's blood and remain raised for 4-10 days. It has been widely recognized that elevated cTnT and cTnI can help identify risks of future major cardiac events (Oscarsson et al., 2004). As a specific marker for myocardial cell injury, cTnI has high myocardial specificity, and has been widely used in ischemic myocardial injury. In recent years, both domestic and foreign scholars have devoted themselves to explorations on cTnI, which has been designated as the gold standard for MI diagnosis abroad (Haggart et al., 2001; Landesberg et al., 2003). Ausset et al demonstrated that troponin I elevation in the first three days after operation was associated with 10-fold higher risk of MACE based on 88 consecutive patients undergoing hip surgery (Ausset et al., 2008). After 6 months of follow-up for 772 patients receiving major noncardiac surgery, Lopez-Jimenez et al. (1997) concluded that patients with elevated cTnT had higher risk of facing cardiac events than those with normal cTnT. Our study also obtained high value for cTnI and cTnT in predicting MACE. In addition, Mochmann et al. (2016) found there was no association between cTn elevation with coronary culprit lesion in acute ischemic stroke patients. However, the pathophysiology of cTn elevation still needs further investigation (Hofmann Bowman and Liao, 2016).

Some large prospective studies have drawn consistent conclusion that CRP could be regarded as a cardiovascular risk factor as traditional risk factors (Kaptoge et al., 2010; Piepoli et al., 2017). In a prospective study enrolled 459 consecutive patients who received kidney transplantation, CRP showed a positive role in predicting cardiac events. (Kruger et al., 2010) However, to our disappointment, we couldn't include CRP in this meta-analysis for only this one suitable study was found. Hs-CRP is an enhanced sensitivity C-reactive protein (CRP) immunoassay with a lowered measurement cutoff, hence, hs-CRP has higher sensitivity than CRP, being measured by laser nephelometry. Moreover, hs-CRP is widely used in clinical practice as a vital inflammatory biomarker. Inflammation is considered to take place in the pathophysiological process of cardiovascular diseases, therefore, hs-CRP, as a vital inflammatory biomarker, would be relevant with cardiac events (Ridker et al., 1997; Owens et al., 2007). In a 15-month follow-up study, increased hs-CRP plasma concentration was reported to be associated with unfavorable prognosis in non-ST elevation acute myocardial infarction (NSTEMI) patients with AF (Pavlovic et al., 2017). Nortamo et al. (2017) reported hs-CRP could predict the risk of new-onset AF in patients with CAD. In a meta-analysis including 83,995 participants, Li et al. (2017) demonstrated that hs-CRP could be significant risk factor in predicting all-cause, cardiovascular mortality. Based on a total of 101 patients undergoing noncardiac surgery, Martins et al. (2011) demonstrated that elevated hs-CRP levels meant increased risk of perioperative acute myocardial infarction. In our meta-analysis, relevant results on the basis of four studies showed that hs-CRP might be regarded as a predictor for MACE in noncardiac surgery patients, and that the higher hs-CRP levels, preoperation or postoperation immediately, the higher risk of developing postoperative MACE.

### Strength and Limitations

To our knowledge, this is the first study to evaluate the relationship between five biomarkers (BNP, NT-proBNP, cTnI, cTnT, and hs-CRP) and MACE. After examinations, we found that all of these biomarkers could be used as the predictors for postoperative MACE, and such findings may have certain clinic value in protecting patients undergoing noncardiac surgery from perioperative MACE. In this meta-analysis, eligible studies involved numerous noncardiac surgeries, such as hip surgery, vascular surgery and kidney transplantation. However, there are some limitations: Firstly, it is impossible for us to incorporate all kinds of noncardiac surgeries in this meta-analysis. Secondly, it would be more persuasive to embrace more studies on the association between biomarker and MACE, but so far, relevant clinical studies were limited. Only 4 studies about hs-CRP were included in this meta-analysis, and we couldn't do a meta-analysis about CRP for only 1 study about CRP was found. We will follow the clinical study progression in this area and rewrite the meta-analysis in the future. In addition, consensus has not been achieved on how to define normal range and on how to use these indicators to classify preoperative risks. Studies on how to utilize perioperative cardiac biomarkers to predict MACE after non cardiac surgery need to be further improved, thus reducing the incidence of MACE after non cardiac surgery.

## Conclusion

In conclusion, BNP, NT-proBNP, cTnI, cTnT and hs-CRP are all effective predictors for cardiovascular risk during noncardiac surgery. The elevation of these five biomarkers all increased the risk of MACE. Therefore, these biomarkers would be helpful for predicting MACE among patients undergoing noncardiac surgery.

## Author Contributions

M-YL contributed to the design of the paper. L-JZ contributed to data collection and paper revision. NL and YL contributed to data collection and analyses, and paper writing. X-TZ contributed to statistical analyses and paper revision.

### Conflict of Interest Statement

The authors declare that the research was conducted in the absence of any commercial or financial relationships that could be construed as a potential conflict of interest.
